# The seasonal variations of allelopathic activity and allelopathic substances in *Brachiaria brizantha*

**DOI:** 10.1186/s40529-015-0105-7

**Published:** 2015-09-19

**Authors:** Ai Kobayashi, Hisashi Kato-Noguchi

**Affiliations:** Department of Applied Biological Science, Faculty of Agriculture, Kagawa University, Miki, Kagawa 761-0795 Japan

**Keywords:** Allelopathy, *Brachiariabrizantha*, Growth inhibitor, Phytotoxicity, Soil additive

## Abstract

**Background:**

Controlling weeds through allelopathy is one strategy to reduce dependency on synthetic herbicides. The plant shoots of the grass *Brachiaria* *brizantha* incorporated into the field soil were found to inhibit the growth of several plant species. We investigated the variations of allelopathic activity and allelopathic substances in *B.* *brizantha* harvested in June, October and January.

**Results:**

All extracts of *B. brizantha* obtained from June, October and January inhibited the root and shoot growth of cress, lettuce, *Phleum pretense* and *Lolium multiflorum* in a concentration dependent manner. However, the inhibitory activity of *B. brizantha* of June and October was greater than that of *B. brizantha* of January. Concentrations of three potent allelopathic active substances, (*6R,9S*)-3-oxo-α-ionol, (*6R,9R*)-3-oxo-α-ionol and 4-ketopinoresinol were also greater in *B. brizantha* of June and October than those in *B. brizantha* of January.

**Conclusion:**

The results suggest that the allelopathic activity and the levels of those allelopathic active substances are greater in *B. brizantha* of June and October than those in *B.* *brizantha* of January. It is possible that *B. brizantha* could be useful for a weed suppressive residue or soil additive materials in the variety of agricultural settings to develop sustainable agriculture options. The effectiveness of *B. brizantha* of June and October as a weed suppressive agent may be greater than that of January.

## Background

Plant allelopathy has been investigated for long time to develop sustainable agricultural systems especially in weed control purposes (Belz 2007; Macías et al. 2007). Plants contain hundreds of secondary metabolites, and some of those compounds showed strong allelopathic activity such as germination and growth inhibitory effects on other plants (Inderjit 1996; Duke et al. 2000; Macías et al. 2007). Some plant materials and residues incorporated into field soil provided excellent weed control ability due to the allelopathic active substances in those materials. Intercropping of allelopathic plants with crop plants also inhibited weed germination and growth owing to their allelopathic property (Weston 1996; Narwal 1999). Therefore, plant allelopathy is probably useful for weed management options in the sustainable agricultural settings (Putnam 1988; Weston 1996; Narwal 1999).

Poaceae species *Brachiaria* *brizantha,* originating in Africa savannas, is introduced into many countries as livestock forage (McGregor et al. 1988). The extracts of *B.* *brizantha* shoots inhibited the seed germination of *Stylosanthes* species (Rodrigues et al. 2012) and seedling growth of cress, lettuce, *Phleum pratense* and *Lolium multiflorum* (Kato-Noguchi et al. 2014). In addition, the plant shoots of *B.* *brizantha* incorporated into the field soil inhibited the growth of several plant species (Martins et al. 2006; Souza et al. 2006). Three allelopathic active substances, (*6R,9S*)-3-oxo-α-ionol, (*6R,9R*)-3-oxo-α-ionol and 4-ketopinoresinol were isolated from *B.* *brizantha* shoots (Kato-Noguchi et al. 2014). Therefore, *B.* *brizantha* shoots may be also useful as soil additives for weed management options in some sustainable agricultural settings because of their allelopathic active substances.

*B.* *brizantha* is a perennial grass, 1–2 m in height, with 2 m deep roots and rhizomes. The plant shoots with long leaf blades grow from spring to autumn and die in winter (Miles et al. 1996; Cook et al. 2005). Therefore, it is important to evaluate the variations of the allelopathic activity and the levels of allelopathic active substance in *B.* *brizantha* for soil additive materials. However, there is no information available about those variations. In the present research, allelopathic activity of *B.* *brizantha* shoots and the level of allelopathic active substances in the shoots were determined in June, October and January.

## Methods

### Plant materials

*Brachiaria* *brizantha* (Hochst. ex A. Rich.) Stapf. (common name; palisade grass, bread grass, syn; *Urochloa brizantha* Hochst. ex A. Rich.) was grown in the research field of National Institute for Agro-Environmental Sciences, Tsukuba, Japan. Shoots of the plants were harvested in June and October 2011 and January 2012, and stored at −20 °C until extraction. Cress (*Lepidum sativum* L.) and lettuce (*Lactuca sativa* L.) were used due to their uniform establishment and sensitivity as a seedling indicator (Kato-Noguchi et al. 2010, 2014). Weed species, *Phleum pratense* L. and *Lolium multiflorum* Lam were also used for bioassay.

### Extraction and bioassay

*B.* *brizantha* (100 g dry weight) shoots were extracted with 1 L of 70 % (v/v) aqueous methanol for 2 days. After filtration with filter paper (No. 2; Toyo, Tokyo, Japan), the residue was extracted again with 1 L methanol for 2 days and filtered, and the two filtrates were combined. An aliquot of the extract (final assay concentration of tested samples corresponded to the extracts obtained from 0.3, 1, 3, 10, 30, 100 and 300 mg dry weight of *B.* *brizantha* shoots per mL) was evaporated to dryness, dissolved in a 0.2 mL of methanol and added to a sheet of filter paper (No. 2) in a 3-cm Petri dish. The methanol was evaporated in a fume hood. Then, the filter paper in the Petri dishes was moistened with 0.8 mL of 0.05 % (v/v) polyoxyethylene sorbitan monolaurate (Tween 20). Ten seeds of cress or lettuce, or 10 seedlings of *P. pratense* or *L. multiflorum* after germination in the darkness at 25 °C for 36–48 h were placed in the Petri dishes. The length of roots and shoots of these seedlings were measured after 48 h of incubation in the darkness at 25 °C. Controls were treated exactly as described above, with the exception that 0.2 mL methanol was used instead of *B.* *brizantha* extracts. Inhibitory activity (%) was determined by the formula: [(control plant length − plant length incubated with extract)/control plant length] × 100. The bioassay was repeated five times using a randomized design with 10 plants for each determination. Significant differences were examined by Duncan’s multiple comparison tests.

### Quantification of allelopathic active substances

*Brachiaria* *decumbens* shoots was extracted as described above and the extracts were concentrated at 40 °C in vacuo to produce an aqueous residue. The aqueous residue was adjusted to pH 7.0 with 1 M phosphate buffer and partitioned three times against an equal volume of ethyl acetate as described by Kato-Noguchi et al. (2014). The ethyl acetate fraction was evaporated to dryness and separated on a column of silica gel (100 g, silica gel 60, 70–230 mesh; Merck), eluted with 20, 30, 40, 50, 60, 70 and 80 % ethyl acetate in *n*-hexane (100 mL per step) and ethyl acetate (100 mL). Allelopathic active substances, (*6R,9R*)-3-oxo-α-ionol and (*6R,9S*)-3-oxo-α-ionol, were obtained by the elution with 70 % ethyl acetate in *n*-hexane on the silica gel column, and 4-ketopinoresinol was obtained by the elution with 80 % ethyl acetate in *n*-hexane on the silica gel column (Kato-Noguchi et al. 2014).

For quantification of (*6R,9R*)-3-oxo-α-ionol and (*6R,9S*)-3-oxo-α-ionol, the fraction obtained with 70 % ethyl acetate in *n*-hexane on the silica gel column was evaporated. The residue was then dissolved in 20 % (v/v) aqueous methanol (2 mL) and loaded onto reverse-phase C_18_ cartridges (YMC Ltd., Kyoto, Japan). The cartridge was eluted with 20, 40, 60 and 80 % (v/v) aqueous methanol (15 mL per step). The active fraction was eluted by 40 % aqueous methanol and evaporated to dryness. The residue was injected into reverse-phase HPLC (4.6 mm i.d. × 250 mm, Inertsil ODS-3, GL Sciences, Osaka, Japan) eluted at a flow rate of 0.8 mL min^−1^ with 55 % aqueous methanol and detected at 220 nm. Retention time of (*6R,9R*)-3-oxo-α-ionol and (*6R,9S*)-3-oxo-α-ionol was 65 and 70 mm, respectively. Quantification of those compounds was performed by measuring their peak areas on the chromatogram of HPLC. The quantification was repeated three times independently with three assays for each determination. Significant differences were examined by Duncan’s multiple comparison tests.

The ^1^H-NMR spectrum of (*6R,9R*)-3-oxo-α-ionol, δ_H_: 1.00 (3H, s), 1.03 (3H, s), 1.24 (3H, d, *J* = 6.8 Hz), 1.94 (3H, d, *J* = 1.0 Hz), 2.05 (1H, d, *J* = 16.6 Hz), 2.40 (1H, d, *J* = 16.6 Hz), 2.67 (1H, d, *J* = 9.3 Hz), 4.27 (1H, m), 5.58 (1H, dd, *J* = 15.1, 8.8 Hz), 5.70 (1H, dd, *J* = 15.2, 5.9 Hz), 5.88 (1H, s). The specific rotation of the compound $\left( {\left[ \alpha \right]^{ 2 5}_{\text{D}} } \right)$ was +210° (*c* 0.03, CH_2_Cl_2_). The ^1^H-NMR spectrum of (*6R,9S*)-3-oxo-α-ionol, δ_H_: 0.98 (3H, s), 1.03 (3H, s), 1.24 (3H, d, *J* = 6.4 Hz), 1.96 (3H, d, *J* = 1.5 Hz), 2.05 (1H, d, *J* = 16.6 Hz), 2.42 (1H, d, *J* = 17.1 Hz), 2.66 (1H, d, *J* = 8.8 Hz), 4.28 (1H, m), 5.55 (1H, dd, *J* = 15.1, 8.8 Hz), 5.69 (1H, dd, *J* = 15.1, 5.9 Hz), 5.89 (1H, s). The specific rotation of the compound $\left( {\left[ \alpha \right]^{ 2 5}_{\text{D}} } \right)$ was +214° (*c* 0.05, CH_2_Cl_2_).

For quantification of 4-ketopinoresinol obtained with 80 % ethyl acetate in *n*-hexane on the silica gel column was evaporated. The residue was then dissolved in 20 % (v/v) aqueous methanol (2 mL) and loaded onto reverse-phase C_18_ cartridges. The cartridge was eluted with 20, 40, 60 and 80 % (v/v) aqueous methanol (15 mL per step). The active fraction was eluted by 40 % aqueous methanol and evaporated to dryness. The residue was injected into reverse-phase HPLC (10 mm i.d. × 50 cm, ODS AQ-325; YMC Ltd.) eluted at a flow rate of 1.5 mL min^−1^ with 40 % aqueous methanol and detected at 220 nm. Retention time of 4-ketopinoresinol was 145 mm and quantification of the compound was performed by measuring their peak areas as described above.

The ^1^H-NMR spectrum of 4-ketopinoresinol, δ_H_: 3.25 (m, 1 H, H1), 3.47 (dd, *J* = 9.2, 3.8 Hz, 1 H, H5), 3.90 (s, 3 H, OCH_3_), 3.92 (s, 3 H, OCH_3_), 4.04 (dd, *J* = 9.4, 4.5 Hz, 1 H, H8a), 4.34 (dd, *J* = 9.3, 6.8 Hz, 1 H, H8b), 5.34 (d, *J* = 4.0 Hz, 1 H, H2), 5.35 (d, *J* = 3.6 Hz, 1 H, H6), 6.8 (m, 2H, aromatic H), 6.9 (m, 4H, aromatic H). The ^13^C NMR spectrum of the compound, δ_C_: 50.1 (C1), 53.5 (C5), 56.2 (OCH_3_), 56.2 (OCH_3_), 72.8 (C8), 83.5 (C2), 84.8 (C6), 107.9 (C2′), 108.2 (C2″), 114.5 (C5″), 114.8 (C5′), 118.1 (C6″), 118.5 (C6′), 131.2(C1′), 132.4 (C1″), 145.4 (C4″), 146.2 (C4′), 146.9 (C3″), 147.1 (C3′), 177.1 (C4). The specific rotation of the compound $\left( {\left[ \alpha \right]^{ 2 5}_{\text{D}} } \right)$ was +5.7° (*c* 0.1, CH_3_OH).

## Results and discussion

**Fig. 1:**
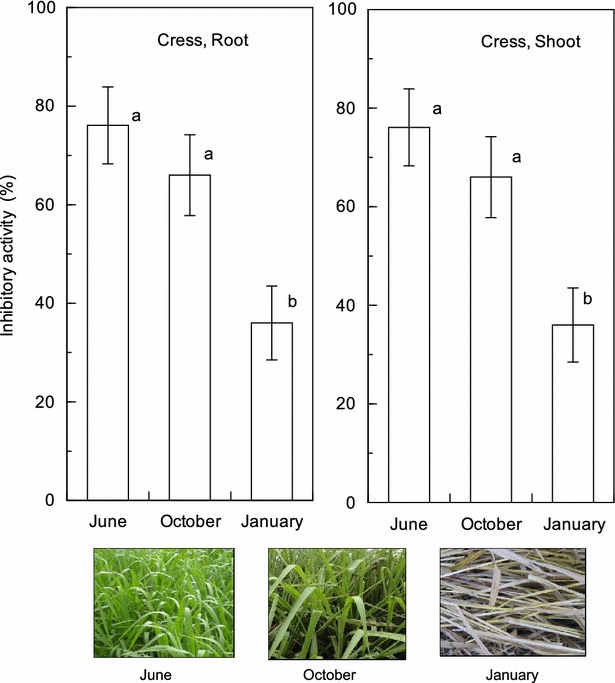
Effects of aqueous methanol extracts of *B. brizantha* shoots harvested in June, October and January on cress root and shoot growth. Concentration of tested sample corresponded to the extract obtained from 10 mg dry weight of *B. brizantha* per mL. Mean ± SE from five independent experiments with ten plants for each determination are shown. The *different letters* in the same groups indicate significant difference (*P* < 0.05) according to Duncan’s multiple comparison tests

**Fig. 2:**
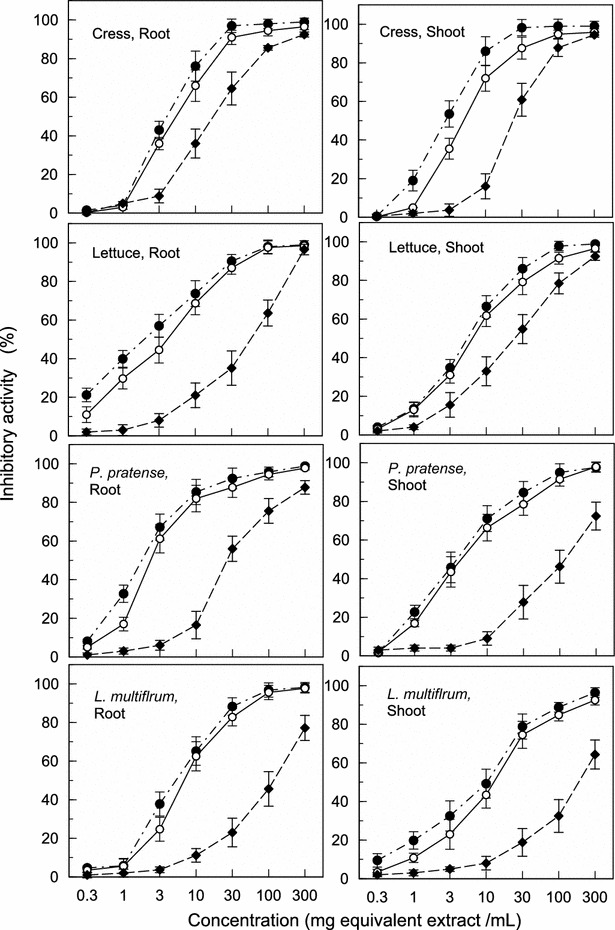
Effects of aqueous methanol extracts of *B. brizantha* shoots harvested in June (*filled circle*), October (*unfilled circle*) and January (*filled diamond*) on root and shoot growth of cress, lettuce, *P. pretense* and *L. multiflorum*. Concentrations of tested samples corresponded to the extracts obtained from 0.3, 1, 3, 10, 30, 100 and 300 mg dry weight of *B. brizantha* per mL. Mean ± SE from five independent experiments with ten plants for each determination are shown

Three extracts obtained from 10 mg *B. brizantha* shoots harvested in June, October and January inhibited the growth of cress roots and shoots (Fig. 1). The inhibitory activity of the June extract on the cress roots and shoots was 76.1 and 79.5 %, respectively, that of the October extract on the cress roots and shoots was 55.7 and 71.5 %, respectively, and that of the January extract on the cress roots and shoots was 35.8 and 15.8 % respectively. Although *B. brizantha* is perennial plant, its shoots re-start to grow around May and die in November and only its underground parts survive after November. The shoots of the plants were growing stage in June and mature stage in October and both shoots were green. The shoots of January had already died and turned brown (Fig. 2). The results indicate that allelopathic activity of *B. brizantha* of June and October was greater than that of January. Thus, green plant materials may be better than dead plant materials for a weed suppressive residue or soil additive materials.

Inhibitory activity of *B. brizantha* shoot extracts obtained from June, October and January was determined by root and shoot growth of cress, lettuce, *P. pretense* and *L. multiflorum* (Fig. 2). All extracts inhibited the growth of the roots and shoots of those test plants with extract-concentration dependent. The concentrations required for 50 % growth inhibition on the roots and shoots of the test plants in the assay (defined as I_50_) were determined by a logistic regression (Y = b + a log_10_X. Y;  % length, X; concentration, a; slope, b; intercept) analysis (Table 1). Comparing those I_50_ values, inhibitory activity of the June extract was 1.1- to 1.8-fold and 4.4- to 27.8-fold greater than that of the October extract and January extract, respectively. These results suggest that all *B. brizantha* extracts have inhibitory effects on both dicotyledonous plants (cress and lettuce) and monocotyledonous weed plants (*P. pretense* and *L. multiflorum*) and the June extract has the greatest inhibitory activity.

**Table 1:** I_50_ of aqueous methanol extracts of *B. brizantha* shoots harvested in June, October and January on root and shoot growth of cress, lettuce, *P. pretense* and *L. multiflorum*

	I_50_ (mg equivalent extract/mL)		
	June	October	January
Cress			
Root	4.5	6.3	19.8
Shoot	2.8	4.9	22.3
Lettuce			
Root	2.5	4.5	55.7
Shoot	6.3	7.9	25.3
*P. pratense*			
Root	1.8	2.5	23.7
Shoot	4.4	4.7	122.5
*L. multiflorum*			
Root	4.9	7.2	123.5
Shoot	9.8	13.8	211.3

**Fig. 3:**
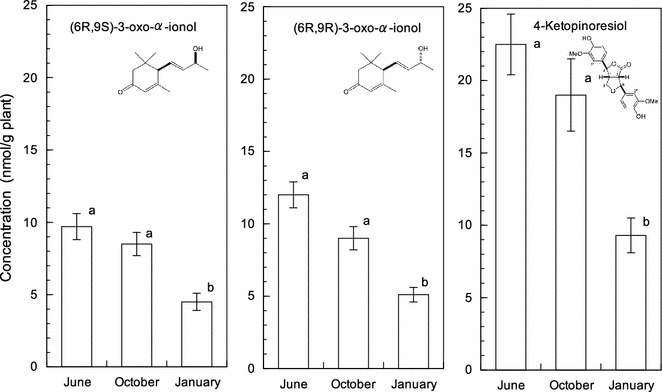
Concentration of (*6R,9S*)-3-oxo-α-ionol, (*6R,9R*)-3-oxo-α-ionol and 4-ketopinoresinol in *B. brizantha* shoots harvested in June, October and January. Mean ± SE from three independent experiments with three quantifications for each determination are shown. The *different letters* in the same groups indicate significant difference (*P* < 0.05) according to Duncan’s multiple comparison tests

The values were determined by a logistic regression analysis after bioassays

(*6R,9S*)-3-oxo-α-ionol, (*6R,9R*)-3-oxo-α-ionol and 4-ketopinoresinol were isolated from *B. brizantha* shoots as three major active substances in *B. brizantha* (Kato-Noguchi et al. 2014). Thus, concentrations of those allelopathic active substances, (*6R,9S*)-3-oxo-α-ionol, (*6R,9R*)-3-oxo-α-ionol and 4-ketopinoresinol were determined in *B. brizantha* shoots of June, October and January (Fig. 3). Those concentrations in the January shoots were significantly lower than those in the June and October shoots. Concentration of (*6R,9S*)-3-oxo-α-ionol, (*6R,9R*)-3-oxo-α-ionol and 4-ketopinoresinol in the June shoots was 2.3-, 2.2- and 2.4-fold greater than that in the January shoots, respectively. This result is consistent with the result of inhibitory activity of the shoot extracts of June, October and January (Table 1) and also indicates that June and October shoots may be better than January shoots for a weed suppressive residue or soil additive materials.

(*6R,9S*)-3-Oxo-α-ionol, (*6R,9R*)-3-oxo-α-ionol and 4-ketopinoresinol inhibited the growth of cress at concentrations greater than 10, 30 and 30 μM, respectively. I_50_ values on cress roots and shoots were 223 and 156 μM for (*6R,9R*)-3-oxo-α-ionol, respectively, and 41.7 and 25.1 μM for (*6R,9S*)-3-oxo-α-ionol, respectively, and 83.5 and 141 μM for 4-ketopinoresinol, respectively (Kato-Noguchi et al. 2014). Therefore, the inhibitory activity of (*6R,9S*)-3-oxo-α-ionol was 5.3 to 6.2- and 2 to 5.6-fold greater than that of (*6R,9R*)-3-oxo-α-ionol and 4-ketopinoresinol, respectively. These results indicate that the growth inhibitory activity of (*6R,9S*)-3-oxo-α-ionol is the greatest, followed by 4-ketopinoresinol and (*6R,9R*)-3-oxo-α-ionol.

3-Oxo-α-ionol was first identified as the aglycone of a glucoside isolated from *Rubus idaeus* (Pabst et al. 1992; D’Abrosca et al. 2004) and was reported to act as allelopathic active substance in *rattail fescue* (Kato-Noguchi et al. 2010). However, the specific biological activities of (*6R,9R*)-3-oxo-α-ionol and (*6R,9S*)-3-oxo-α-ionol were first reported by Kato-Noguchi et al. (2014). 4-Ketopinoresinol was first isolated from *Aegilops ovate* as germination inhibitor (Lavie et al. 1974; Cooper et al. 1977), but the information on the biological activity of the compound is very limited.

Phytotoxic active substances in plants can be released into the soil, either by exudates from living plant tissues or by decomposition of plant residues, and act as allelopathic active substances which inhibit seed germination, seedling establishment and plant growth (Bais et al. 2006; Bonanomi et al. 2006; Belz 2007). The present results showed that *B. brizantha* inhibited root and shoot growth of four test plant species including weed plants (*P. pretense* and *L. multiflorum*) in a concentration dependent manner. Thus, *B. brizantha* could be useful for a weed suppressive residue or soil additive materials in the variety of agricultural settings to reduce dependency on synthetic herbicides. Natural products are considered to be more environmentally benign than most synthetic herbicides. Their environmental half-lives can be expected to be shorter, and the fact that they have been part of the natural environment suggests a lesser demand for regulatory measures (Duke et al. 2000). In addition, *B. brizantha* has been reported to have a potential to enhance mineral nutrients (Souza et al. 2006). Further studies are required to determine the effectiveness of *B. brizantha* on weeds in the field conditions.

## Conclusion

The extracts of *B. brizantha* shoots harvested in June, October and January inhibited root and shoot growth of cress, lettuce, *Phleum pretense* and *Lolium multiflorum* in a concentration dependent manner. However, the inhibitory activity of *B. brizantha* of June and October was greater than that of *B. brizantha* of January. Concentrations of three allelopathic active substances, (*6R,9S*)-3-oxo-α-ionol, (*6R,9R*)-3-oxo-α-ionol and 4-ketopinoresinol were also greater in *B. brizantha* shoots of June and October than those in *B. brizantha* shoots of January. Therefore, *B. brizantha* could be useful for a weed suppressive residue or soil additive materials in the variety of agricultural settings to develop sustainable agriculture options due to those allelopathic active substances. The effectiveness of *B. brizantha* of June and October as a weed suppressive agent may be greater than that of January.

## Abbreviations

I_50_: concentrations required for 50 % growth inhibition
